# Potential protective regulatory effects on radiation−induced esophageal injury in TUT4^−/−^ mice

**DOI:** 10.3389/fonc.2025.1600597

**Published:** 2025-08-26

**Authors:** Huiwen Ren, Wei Li, Zhigang Fan, Jianlin Wang, Zhiqiang Sun, Renhua Huang, Judong Luo, Bo Gao

**Affiliations:** ^1^ Department of Radiotherapy, Tongji Hospital, School of Medicine, Tongji University, Shanghai, China; ^2^ Clinical Nutrition Department of The Tenth People’s Hospital, Tongji University, Shanghai, China; ^3^ Department of Oncology, 3201 Hospital of Xi’an Jiaotong University Health Science Center, Shanghai, China; ^4^ Department of Radiotherapy, The Second People’s Hospital of Changzhou, the Third Affiliated Hospital of Nanjing Medical University, Changzhou, China; ^5^ Department of Radiation, Ren Ji Hospital, Shanghai Jiao Tong University School of Medicine, Shanghai, China; ^6^ Department of Rheumatology and Immunology, The Second People's Hospital of Changzhou, the Third Affilitated Hosptial of Nanjing Medical University, Changzhou, China

**Keywords:** esophageal tissue, radioprotection, TUT4, radiation-induced esophageal injury, radioactivity

## Abstract

**Introduction:**

Terminal uridyl transferase 4 (TUT4), a nucleotide transferase that modifies miRNA sequences, plays a critical role in regulating miRNA target interactions and function. However, its involvement in radiation-induced esophageal injury remains poorly understood.

**Methods:**

To investigate this, we performed computational analysis of RNA-seq data from irradiated esophageal tissues of wild-type and TUT4-knockout (TUT4^–/–^) mice, identifying 53 differentially expressed mRNAs (DEmRNAs), of which 30 were upregulated and 23 downregulated.

**Results:**

Gene Ontology and Kyoto Encyclopedia of Genes and Genomes enrichment analyses revealed that these DEmRNAs were significantly associated with biological processes including lipid metabolism, fatty acid metabolism, proteolysis, and broader metabolic functions. Notably, DEmRNAs in TUT4^–/–^ esophageal tissues showed marked enrichment in the renin–angiotensin system and peroxisome proliferator-activated receptor signaling pathways, implicating their potential roles in the pathogenesis of radiation-induced esophageal injury. In addition, we identified a regulatory axis in which a long non-coding RNA competes with miR-182 to modulate the competing endogenous RNA network governing TUT4 target genes. Collectively, our transcriptomic analysis offers novel mechanistic insights into how TUT4 may confer protection against radiation-induced damage in esophageal tissues.

## 1 Introduction

Radiotherapy remains a cornerstone in cancer treatment, often involving high doses of ionizing radiation. However, radiation-induced esophageal injury—a non-specific inflammatory response in the esophageal mucosa—is a frequent adverse effect, particularly among patients undergoing radiotherapy for tumors in the neck, chest, or mediastinum. This condition manifests as acute radiation esophagitis (ARE) within three months of initiating treatment, or late radiation esophagitis (LRE) if symptoms appear thereafter. ARE typically develops rapidly, most often within 2–3 weeks of the initial radiation dose. Radiation exposure disrupts esophageal tissue integrity, generating large quantities of oxygen-derived free radicals. These reactive species damage cell membranes by reducing fluidity, increasing permeability, inducing mitochondrial swelling, and promoting lysosomal enzyme release. This cascade culminates in cellular injury and triggers an inflammatory response. Clinically, ARE may initially present as a foreign body sensation during swallowing, which can progress to odynophagia, dysphagia, or persistent retrosternal pain unrelated to deglutition.

In contrast, LRE generally manifests more than three months after radiotherapy, with some cases presenting up to a year later. Pathologically, LRE is characterized by esophageal fibrosis, damage to the muscularis propria, and possible neural injury. These changes may lead to dysphagia secondary to luminal stenosis, impaired motility, or chronic ulceration. Severe cases may be complicated by life-threatening events such as esophageal perforation, esophagotracheal fistula, or aorto-esophageal fistula (1).

Notably, radiation-induced esophageal injury often necessitates treatment interruption and, in some cases, treatment discontinuation (2–4). Despite its clinical significance, no pharmacologic agent is currently approved for its prevention or treatment. Nevertheless, adjunctive therapies—such as amifostine (5) or soy isoflavone supplementation (6)—have demonstrated efficacy in reducing the incidence and severity of esophageal injury during thoracic radiotherapy.

RNA-dependent nucleic acid transferases, members of the β superfamily of DNA polymerases, include several enzymes such as mRNA poly(A) polymerase, poly(U) polymerase, CCA-adding enzyme, and terminal uridylyl transferase (TUTase) (7, 8). In mammals, the TUT family comprises three major uridyl transferases—TUT4 (Zcchc11), TUT7 (Zcchc6), and TUT1—which catalyze template-independent uridine addition to RNA molecules during post-maturation processing. Recent studies have revealed that mRNA poly(A) tails can recruit TUT4 for uridylation of specific mRNAs. Importantly, terminal uridylation functions as a regulatory signal modulating mRNA stability and expression, while exerting broader functional effects on microRNAs (miRNAs). Initially identified as key regulators of miRNA biogenesis in embryonic stem cells, TUT family enzymes—particularly TUT4—interact with the RNA-binding protein Lin28 to selectively uridylate precursor miRNAs such as pre-let-7 (9). TUT4 and related enzymes exhibit spatiotemporal and tissue-specific activity, catalyzing uridylation of diverse miRNAs including let-7, miR-10a/b, miR-26a/b, and miR-100 (7, 8, 10). Given the small size of miRNAs (~22 nucleotides), 3’-terminal uridylation can significantly alter target specificity and downstream gene regulation. For instance, under normal conditions, miR-26 represses IL-6 to modulate inflammatory responses. However, in TUT4-overexpressing cells, miR-26 variants bearing 1–3 uridine residues fail to bind the IL-6 3′ UTR, thereby impairing IL-6 repression (11).

Our prior work demonstrated elevated TUT4 expression in esophageal epithelial cells following exposure to ionizing radiation. Notably, TUT4 mediates radiation-induced esophageal injury through uridylation of miR-132/212 (12, 13). **Table 1**: Top 10 downregulated mRNAs in irradiated TUT4^–/–^esophageal tissues

**Table 1:** Top 10 downregulated mRNAs in irradiated TUT4^–/–^esophageal tissues.

mRNA	log2FoldChange	*P* value	Regulation direction
2310057J18Rik	-22.03360781	1.73E-08	Down
Zcchc11	-1.156692739	1.11E-06	Down
Lipf	-10.85254755	7.64E-05	Down
Sprr2d	-1.228247105	0.000100817	Down
Gm42031	-3.53631731	0.000377563	Down
Uox	-1.572757057	0.001727824	Down
Cma1	-1.568589843	0.003694728	Down
Calca	-5.592707297	0.006960912	Down
Idi2	-1.412512854	0.00736377	Down
Sprr2b	-1.855594047	0.00752417	Down

pathways related to DNA replication, ferroptosis, and cell cycle regulation, implicating these processes in radiation-induced tissue injury (14). We further developed TUT4^−/−^ mice, which exhibited more severe radiation-induced esophageal injury compared to wild-type (WT) controls. Early histopathological changes included capillary congestion, epithelial thinning, and inflammatory cell infiltration, consistent with prior reports (6, 15). At later stages, TUT4−/− mice displayed muscle layer degeneration, fibrosis, and impaired tissue repair. To clarify the molecular mechanisms underlying TUT4-mediated injury, we performed RNA sequencing and downstream bioinformatics analyses. Gene Ontology (GO) and Kyoto Encyclopedia of Genes and Genomes (KEGG) enrichment identified the peroxisome proliferator-activated receptor (PPAR) and renin–angiotensin system (RAS) pathways as significantly dysregulated in TUT4^−/−^ esophageal tissues. These pathways may contribute to the pathogenesis of radiation-induced injury. We further propose that TUT4-dependent uridylation of miR-182 alters its regulatory activity on target genes, thereby modulating cellular radiosensitivity.

## Methods

### Animals and treatments

Female C57BL/6 mice (6–8 weeks old; 18–22 g) and isogenic TUT4^−/−^ mice were procured from Gempharmatech Co., Ltd. (Suzhou, China). All experimental procedures adhered to protocols approved by the Animal Care and Use Committee of Soochow University (Approval No. SUDA20221024A02), and the study complied with ARRIVE 2.0 guidelines. Mice were housed in individually ventilated cages under a 12-hour light/dark cycle at 22°C with controlled humidity and ad libitum access to food and water. For irradiation, mice were anesthetized with an intraperitoneal injection of 3.6% chloral hydrate (1 mL/100 g body weight) and immobilized using adhesive tape. A lead shield (1 cm × 2 cm) was employed to localize the radiation area. Esophageal exposure involved a single dose of 30 Gy administered at 2 Gy/min using a 6-MV X-ray linear accelerator (Clinic 2100EX, Varian Medical Systems, CA). Wild-type mice (n = 6) served as controls, while TUT4 knockout mice (n = 6) comprised the experimental group. Daily food intake and body weight were recorded post-irradiation. Fourteen days after treatment, all mice were euthanized, and esophageal tissues were collected for further analysis.

### Hematoxylin and eosin staining

At weekly intervals post-irradiation, three mice from each group were sacrificed for histological assessment. Esophageal tissues were fixed overnight in 4% paraformaldehyde at 4°C, paraffin-embedded, sectioned (3 μm), deparaffinized, and stained with hematoxylin and eosin (H&E) to evaluate histopathological changes and inflammatory infiltration.

### RNA isolation and library preparation

Total RNA was extracted using the mirVana™ miRNA Isolation Kit (Ambion) following the manufacturer’s protocol. RNA integrity was verified using the Agilent 2100 Bioanalyzer (Agilent Technologies, Santa Clara, CA), and only samples with RIN >7 were used for downstream analysis. Libraries were prepared using the TruSeq Stranded Total RNA with Ribo-Zero Gold Kit (Illumina) and sequenced on an Illumina HiSeq™ 2500 platform (or equivalent) to generate 150/125 bp paired-end reads.

### Data preprocessing and genomic alignment

Raw sequencing data in FASTQ format were processed using Trimmomatic to remove adapters and low-quality bases, including those containing ambiguous nucleotides. High-quality reads were aligned to the reference mouse genome using HISAT2, a splice-aware aligner optimized for high-throughput data. Alignment quality was evaluated at both genome-wide and gene-specific levels to ensure data integrity.

### Transcript splicing, lncRNA prediction, and gene quantification

Transcript assembly and splice variant reconstruction were performed using StringTie. Candidate lncRNAs were identified by comparing assembled transcripts with reference annotations via Cuffcompare. Transcripts with coding potential were excluded using (16), Pfam (17), and PLEK (18), yielding a set of predicted lncRNAs.

Sequencing reads from each sample were aligned to reference mRNA, known lncRNA, and predicted lncRNA sequences using Bowtie2. Transcript quantification was conducted with eXpress, providing FPKM and raw count values for each gene.

### Differential expression analysis

Normalization of raw counts was performed using the estimateSizeFactors function in the DESeq R package. Differential expression analysis was conducted using the biotest function, which computed P values and log_2_(fold change). Transcripts with *P* < 0.05 and |log_2_(fold change)| > 1 were considered significantly differentially expressed.

### LncRNA–miRNA and miRNA–mRNA co-expression networks

A lncRNA–miRNA co-expression network was constructed based on normalized transcript signal intensities, incorporating the top 300 miRNA–lncRNA interaction pairs with the lowest *P* values. A parallel miRNA–mRNA co-expression network was generated using the same criteria.

### GO and KEGG enrichment analyses

DEGs (*P* < 0.05; fold change > 2) were subjected to Gene Ontology (GO) and KEGG pathway enrichment analyses. GO enrichment covered biological processes (BP), cellular components (CC), and molecular functions (MF). KEGG pathway analysis was performed using a hypergeometric distribution test to identify significant functional associations. The KEGG database was used for systematic annotation and interpretation of gene functions.

### Statistical analysis

All quantitative data are presented as mean ± standard deviation (SD). Group comparisons were performed using two-sided Student’s t-tests, with *P* < 0.05 considered statistically significant.

## Results

### Radiation-induced esophageal injury in mouse models

To investigate TUT4’s function in radiation-induced esophagitis, wild-type (WT) and TUT4^−/−^ mice received localized 30 Gy esophageal irradiation.

**Figure 1:**
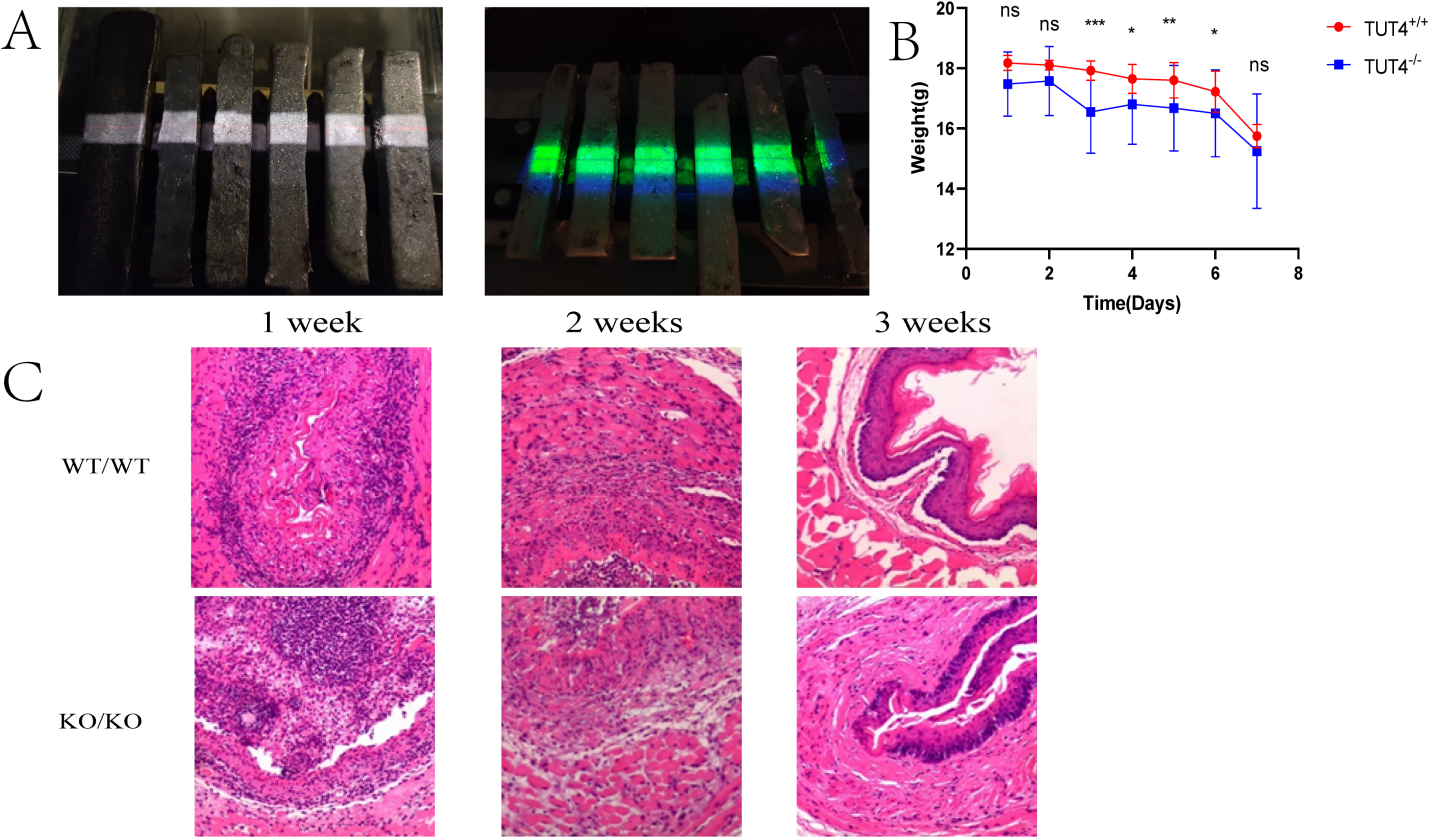
Radiation-induced esophageal injury in mouse models. **(A)** Mouse models of radiation esophagitis. **(B)** Daily body weight of the mice in the first week after irradiation. **(C)** Representative H&E staining of the esophagus of wild-type and knockout mice after irradiation.

At 7 days post-irradiation, TUT4^–/–^ mice exhibited significantly greater weight loss than WT controls (**Figures 1A, B**). Although hematoxylin and eosin (H&E) staining revealed inflammatory responses and granulation tissue formation across all irradiated groups, intergroup differences in inflammation severity were not statistically significant. However, widespread necrosis and epithelial detachment were observed, affecting the squamous, prickle cell, and basal layers, accompanied by architectural disruption and pronounced neutrophilic infiltration (**Figure 1C**). By day 14, intergroup pathological differences became more evident. H&E staining revealed capillary congestion, epithelial thinning, and severe neutrophil- and lymphocyte-dominated inflammation in irradiated tissues (**Figure 1C**). Lesions were more severe in the TUT4^–/–^ group. At 3 weeks post-irradiation, reparative changes such as epithelial hyperplasia and collagen deposition were noted. WT mice exhibited muscular layer edema and intact mucosal, submucosal, and epithelial regeneration, whereas TUT4^–/–^ mice demonstrated muscular degeneration, fibrous hyperplasia, and impaired tissue repair (**Figure 1C**). HE staining of esophageal tissues was performed for quantitative histopathological examination (19) (**Table 2**).

**Table 2:** Evaluation of esophageal tissue damage and infiltrate inflammatory cells in mice of each group.

Group	7d(n=2)			14d(n=2)			21d(n=2)		
	Damage	Neutrophil	Total score	Damage	Neutrophil	Total score	Damage	Neutrophil	Total score
TUT4^+/+^mice	6	6	12	9	8	17	0	0	0
TUT4^-/-^mice	6	6	12	9	6	15	0	0	0

### Expression and correlation of samples

The box plot indicates the distribution of expression data in all samples.

**Figure 2:**
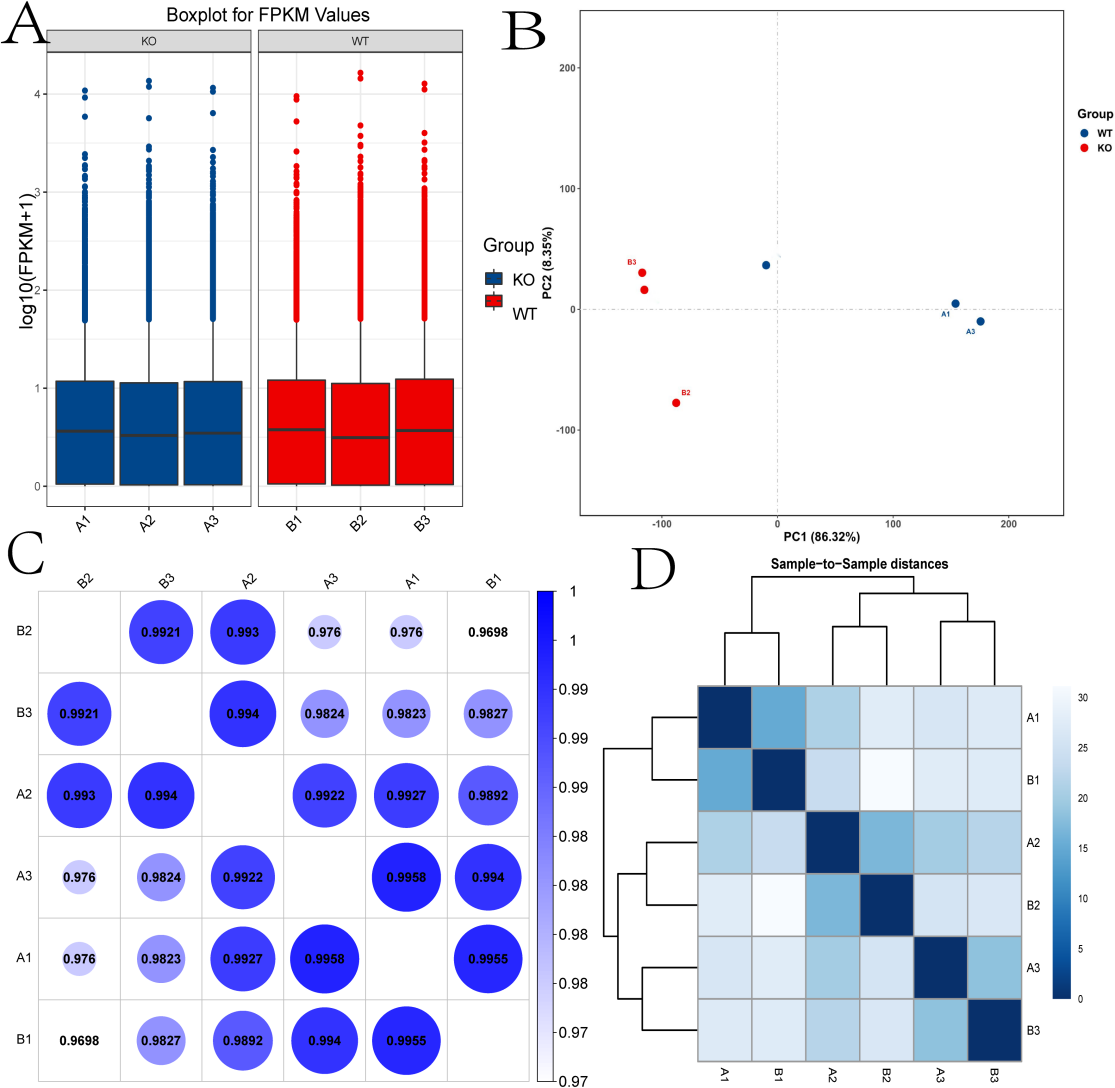
Expression and correlation of the samples. **(A)** Box plot showing the distribution of expression data across all samples. **(B)** Principal component analysis (PCA). **(C)** The correlations among samples were analyzed by Pearson correlation. **(D)** The gene expression cluster diagram of the samples. KO, TUT4^–/–^ mouse group; WT, Wide-Type mouse group.

Box plot analysis of expression data across samples showed high consistency after normalization, indicating minimal batch effects or systematic bias (**Figure 2A**). Pearson correlation analysis of mRNA expression levels confirmed strong intra-group similarity, with the highest correlation observed between samples A1 and A3 (r = 0.9958; **Figure 2B**). Principal component analysis (PCA) revealed a clear transcriptomic divergence between WT and TUT4^–/–^ groups, with red and blue data points representing WT and knockout mice, respectively (**Figure 2C**). Hierarchical clustering further demonstrated distinct groupings based on expression profiles (**Figure 2D**).

### Differentially expressed mRNAs

Differentially expressed mRNAs (DEmRNAs) between WT and TUT4^–/–^ samples were identified using volcano plot analysis.

**Figure 3:**
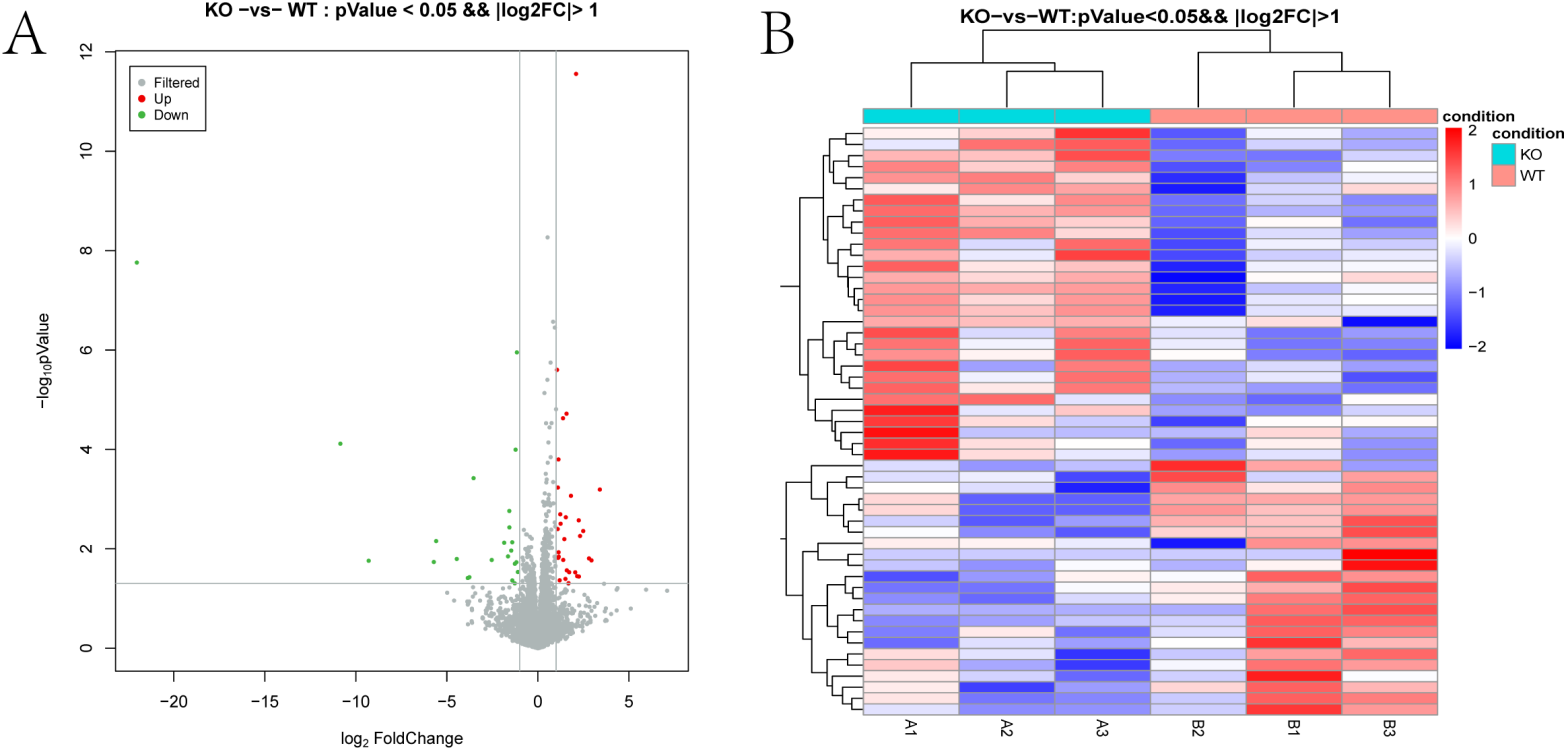
Differentially expressed mRNAs. **(A)** Volcano plot of DEG expression levels between the TUT4 ^–/–^ and TUT4 ^+/+^ groups. The red and green dots in the plot represent the differentially expressed mRNAs with statistical significance. **(B)** Heatmap hierarchical clustering analysis of DEGs in the TUT4 ^–/–^ and TUT4 ^+/+^ group.

Applying a significance threshold of *P* < 0.05 and |log_2_FC| > 1.0, we identified 53 DEmRNAs—30 upregulated and 23 downregulated in the knockout group. In the volcano plot (**Figure 3A**), red and green points represent significantly up- and down-regulated transcripts, respectively. **Tables 1**, **3** list the top ten most up- and downregulated genes. Hierarchical clustering of these DEmRNAs reliably segregated WT and TUT4^–/–^ samples (**Figure 3B**).

**Table 3:** Top 10 upregulated mRNAs in irradiated TUT4^–/–^ esophageal tissues.

mRNA	log2FoldChange	*P* value	Regulation direction
Srp54b	2.094547556	2.76E-12	Up
Thrsp	1.050418106	2.49E-06	Up
Itih4	1.570260638	1.91E-05	Up
Wdfy1	1.376902347	2.35E-05	Up
Cryab	1.12729353	0.000158583	Up
Zbtb16	1.094291321	0.000582251	Up
Ucp1	3.399990553	0.000639038	Up
Gys2	1.81274346	0.000854616	Up
Chil3	1.227073543	0.002010726	Up
Scd1	1.531749594	0.002310168	Up

### Functional annotation of DEmRNAs

To functionally annotate the DEmRNAs, we conducted Gene Ontology (GO) and Kyoto Encyclopedia of Genes and Genomes (KEGG) enrichment analyses.

**Figure 4:**
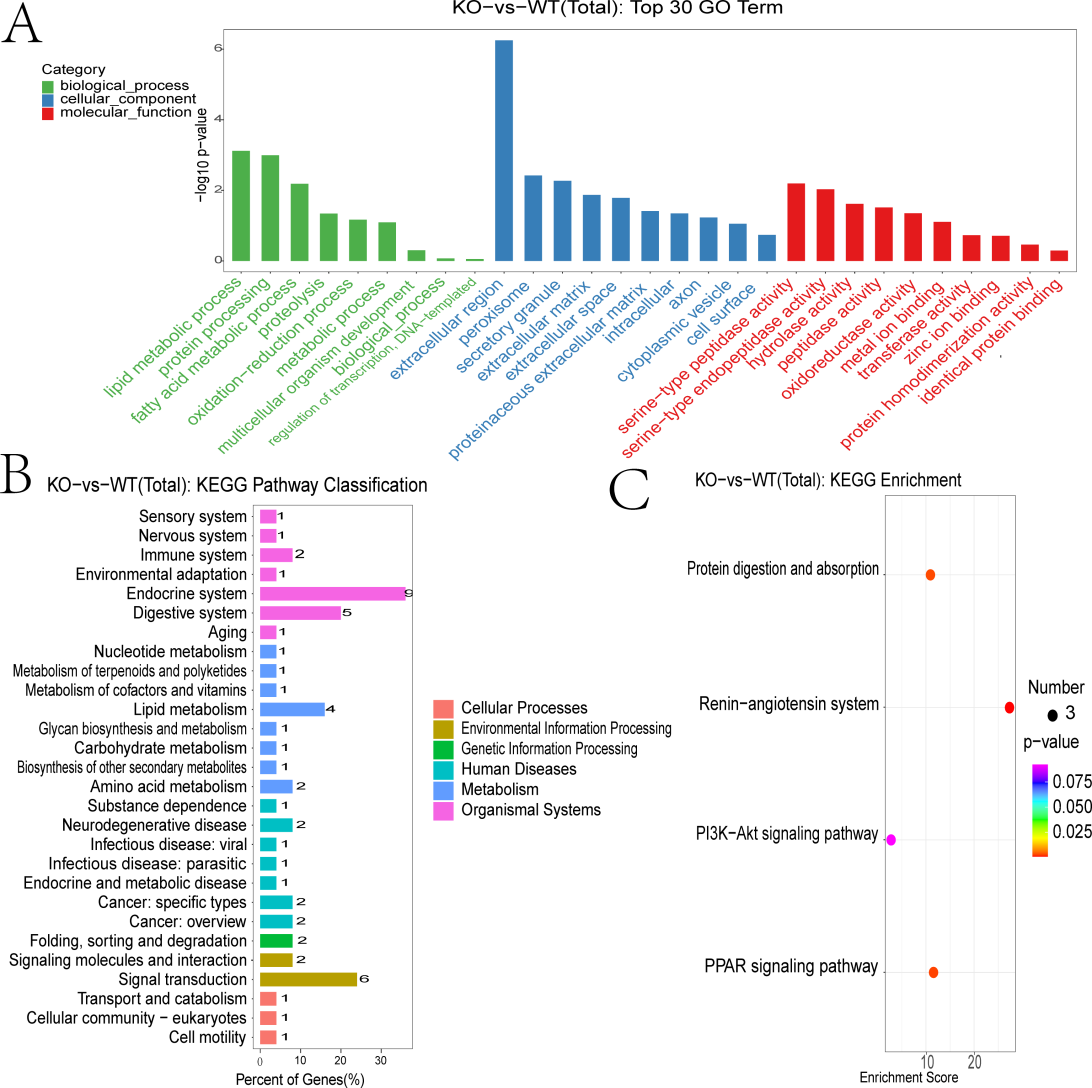
Functional annotation of differentially expressed mRNAs. **(A)** GO enrichment analysis of DEGs showing biological process (BP) (marked in green), cellular component (CC) (marked in blue), and molecular function (MF)(marked in red). **(B)** KEGG enrichment analysis of DEGs. **(C)** KEGG pathway classification of DEGs.

GO biological process (BP) terms showed significant enrichment in lipid metabolic processes, including fatty acid metabolism and proteolysis (**Figure 4A**). Cellular component (CC) terms were enriched for extracellular regions, peroxisomes, and extracellular matrix structures. Molecular function (MF) analysis highlighted serine-type peptidase and hydrolase activities. KEGG pathway analysis further revealed that DEmRNAs were enriched in pathways related to immune, nervous, and digestive systems. Disease associations included viral infections, endocrine and metabolic disorders, and cancer. Enriched environmental information processing pathways encompassed signal transduction and signaling molecule interactions (**Figure 4B**).

DEGs were subjected to the KEGG pathway enrichment analysis, and pathways involving three or more DEGs were visualized (**Figure 4C**). Key enriched pathways included protein digestion and absorption, RAS signaling, PI3K-Akt signaling, and PPAR signaling. Genes associated with the RAS pathway included CMA1, CPA3, THOP1, and CTSG (**Supplementary Figure 1**). Those involved in PPAR signaling included SCD-1, ME1, Thiolase B, UCP-1, ILK, UBC, PEPCK, and GyK (**Supplementary Figure 2**).

### Construction of the ceRNA regulatory network

**Figure 5:**
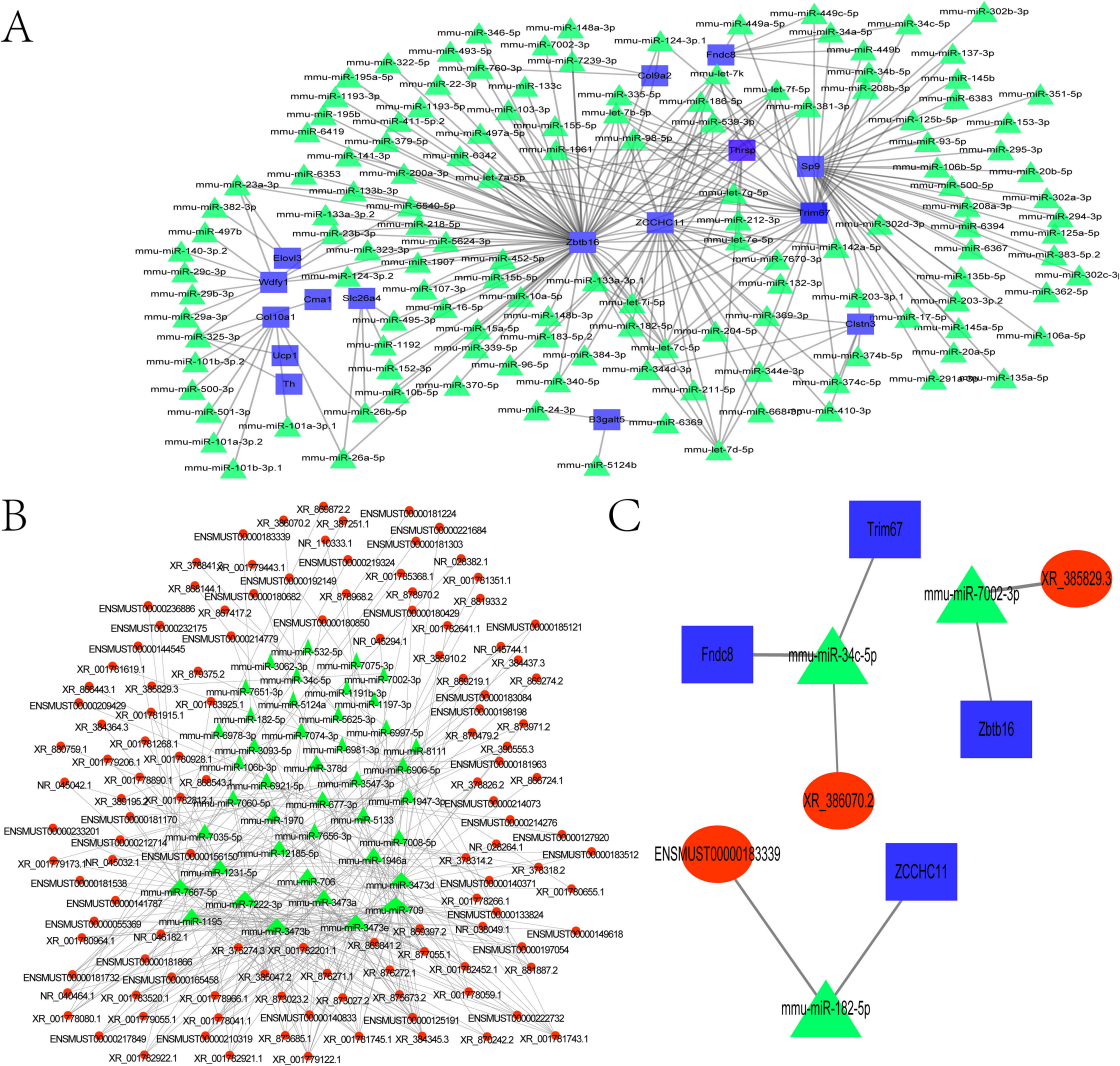
Construction of the ceRNA regulatory network. **(A)** Network of differentially expressed miRNAs and their potential target genes. **(B)** Coexpression network of differentially expressed miRNAs and lncRNAs. **(C)** mRNA–miRNA–lncRNA regulatory network. The mRNAs are marked in blue, the lncRNAs are marked in red, and the miRNA are marked in green.

To investigate potential miRNA functions, the 53 DEGs (30 upregulated, 23 downregulated) were queried against the TargetScan database to predict miRNA–mRNA interactions. This enabled construction of a miRNA–target gene interaction network. From this, the top 300 mRNA–miRNA interaction pairs with the lowest *P* values were identified. Notable predicted target genes included Zbtb16, Zcchc11, Elovl3, Wdfy1, Slc26a4, Cma1, Col10a1, Ucp1, Th, B3galt5, Clstn3, Sp9, Fndc8, and Col9a2 (**Figure 5A**; **Supplementary Table 1**). We constructed the lncRNA–miRNA target interaction network using the R network package. The 300 lncRNA–miRNA interaction pairs with the smallest *P* values were extracted by *P* value sorting (**Figure 5B** and **Supplementary Table 2**).

From the mRNA-miRNA interaction network, we extracted the top 300 most significant pairs (sorted by ascending *P*-value). Potential target genes included *Zbtb16*, *Zcchc11*, *Elovl3*, *Wdfy1*, *Slc26a4*, *Cma1*, *Col10a1*, *Ucp1*, *Th*, *B3galt5*, *Clstn3*, *Sp9*, *Fndc8*, and *Col9a2* (**Figure 5A**; **Supplementary Table 1**). Using the R network package, a lncRNA–miRNA interaction network was similarly constructed. The top 300 interaction pairs were selected based on ascending *P* value ranking (**Figure 5B**; **Supplementary Table 2**). Both networks were integrated using Cytoscape, an open-source platform for biomolecular interaction visualization, in which nodes represent molecules (e.g., genes, proteins) and edges denote their interactions. The merged network of mRNA–miRNA and lncRNA–miRNA interactions was further combined with the ceRNA regulatory network (**Figure 5C**), identifying three significant interaction pairs (**Table 4**).

**Table 4:** Interaction pairs in the ceRNA network.

lncRNA	miRNA	mRNA
ENSMUST00000183339	mmu-miR-182-5p	Zcchc11
XR-386070.2	mmu-miR-34c-5p	Trim67
XR-386070.2	mmu-miR-34c-5p	Fndc8
XR-385829.3	mmu-miR-7002-3p	Zbtb16

Among these, TUT4 was found to bind miR-182, potentially modulating radiation-induced antioxidant responses and enhancing radiotherapy efficacy (20). TRIM67 demonstrated antitumor activity by inhibiting colorectal cancer initiation and progression via p53 activation (21).

## Discussion

In recent decades, thoracic radiation therapy has become a mainstay in the multidisciplinary treatment of breast cancer, lung cancer, Hodgkin’s lymphoma, and other thoracic malignancies, with more than half of patients receiving it (22). Its primary objective is to maximize tumoricidal effects while minimizing collateral damage to surrounding healthy tissues (23). However, radiation esophagitis remains a common and debilitating complication, arising from radiotherapy alone or in combination with chemotherapy. It compromises patient quality of life, restricts radiation dosing, and can precipitate treatment interruptions that reduce therapeutic efficacy (24–26). Clinically, patients often present with dysphagia, odynophagia, nausea, anorexia, or retrosternal burning pain (27). Despite its clinical relevance, the pathophysiological mechanisms underlying radiation-induced esophageal injury remain poorly defined. This study aimed to elucidate the molecular basis of radiation esophagitis, with a specific focus on the role of TUT4. Using transcriptomic sequencing and bioinformatic analysis, we delineated regulatory networks involving mRNAs, lncRNAs, and miRNAs. Subsequent genomic enrichment analysis identified significantly enriched pathways, allowing construction of a comprehensive KEGG network map.

Previous studies using irradiated rat esophageal tissue identified 27 differentially expressed miRNAs (7 downregulated, 20 upregulated) through RNA sequencing, implicating these miRNAs in key biological processes such as cell migration, proliferation, and lipid metabolism. Additionally, 197 differentially expressed circRNAs (87 upregulated, 110 downregulated) were detected. Functional enrichment analysis associated these circRNAs with exosomes, adhesion sites, and notably, sphingolipid metabolic processes (28).

To probe the mechanistic role of TUT4 in radiation-induced esophageal injury, we compared wild-type and TUT4 knockout (TUT4^–/–^) mice subjected to thoracic irradiation. Esophageal tissues were collected at weeks 1, 2, and 3 post-irradiation and analyzed by H&E staining. Findings revealed vascular congestion, squamous epithelial thinning, and extensive inflammatory infiltration—dominated by neutrophils and lymphocytes. Notably, TUT4^–/–^ mice exhibited markedly exacerbated tissue damage, peaking at week 2 post-radiotherapy.

To identify molecular drivers of radiosensitivity and potential therapeutic targets, we performed RNA-seq analysis comparing irradiated esophageal tissues from wild-type and TUT4^–/–^ mice.

Differential expression analysis revealed 53 DEmRNAs (30 upregulated, 23 downregulated). Functional annotation and KEGG pathway analyses indicated significant enrichment in lipid metabolism, fatty acid metabolism, proteolysis, and metabolic regulation. While the cytotoxic effect of radiotherapy is classically attributed to DNA damage, it also induces lipid oxidation. Thus, targeting lipid peroxidation may represent a novel strategy to enhance radiosensitivity (29, 30).

Lipid metabolism is a critical regulator of oncogenic signaling, metastasis, and therapy resistance (31, 32). Notably, radiation has been shown to downregulate lipid metabolism pathways in skin, leading to reduced adipose tissue and altered lipid profiles, suggesting a radioprotective role for tissue lipids (31). We hypothesize that a similar protective mechanism may underlie TUT4-mediated defense against esophageal radiation injury. Our findings show significant modulation of lipid metabolism-related mRNAs during irradiation, implicating lipid metabolism as a key mediator of TUT4’s protective effects. While not all lipid pathways are necessarily TUT4-dependent, the results position lipid metabolism as a promising target for mitigating radiation esophagitis. Further enrichment analysis of TUT4^–/–^ DEGs highlighted involvement of additional pathways, including protein digestion, RAS signaling, PI3K-Akt signaling, and PPAR signaling. The PPAR pathway, often hyperactivated in colorectal cancer, promotes tumorigenesis, while its inhibition suppresses tumor growth and induces apoptosis (33). Importantly, PPAR signaling also contributes to radioresistance in colorectal cancer, with fatty acid metabolism playing a key modulatory role (34).

Tumor cells inhabit highly vascularized microenvironments and secrete pro-angiogenic factors such as VEGF (35). The RAS pathway, primarily involved in cardiovascular and electrolyte regulation has emerged as a therapeutic target in oncology (36). Angiotensin receptor blockers (ARBs) and angiotensin-converting enzyme inhibitors (ACEIs) inhibit tumor angiogenesis, remodel the extracellular matrix, and modulate the hypoxic tumor microenvironment. For instance, the ACEI captopril inhibits tumor angiogenesis and suppresses hepatic metastasis (37). ARBs and ACEIs have shown potential as adjuvants in chemoradiotherapy, enhancing antitumor efficacy (38). Preclinical models also demonstrate ARB-mediated suppression of VEGF signaling and inhibition of pancreatic cancer proliferation (36). RAS blockers further reduce lung cancer metastasis and augment responses to radiotherapy and chemotherapy (39). Our findings show that RAS and PPAR signaling pathways are significantly enriched in TUT4^–/–^ mice, suggesting that TUT4 modulates these pathways to confer protection against radiation-induced esophageal injury. We propose that TUT4 may function downstream of RAS signaling, positioning it as a putative molecular target of RAS-inhibiting therapies for esophageal radioprotection.

To further characterize the regulatory role of TUT4, we constructed ceRNA networks using Cytoscape. Notably, TUT4-mediated uridylation plays a critical role in maintaining genomic stability during early radiotherapy by regulating DNA damage response and repair pathways. Our data suggest that TUT4 protects normal esophageal cells from radiation injury. Among the differentially expressed transcripts related to radiosensitivity, TRIM67 emerged as a key modulator of apoptosis, cell cycle arrest, DNA repair, and senescence. It also suppresses colorectal tumorigenesis through activation of the p53 pathway (21). Furthermore, analysis of The Cancer Genome Atlas by Lin et al. revealed significant upregulation of the miR-182/96/183 cluster following radiotherapy, with miR-182 being the most elevated. Overexpression of miR-182-5p enhances radiosensitivity in head and neck squamous cell carcinoma by elevating intracellular ROS levels and modulating radiation-induced antioxidant responses (20).

By performing RNA-seq on TUT4^–/–^ and wild-type esophageal tissues, we identified key DEGs implicated in radiation-induced injury. Functional analyses highlighted lipid metabolism, RAS signaling, and PPAR signaling as central pathways in TUT4-mediated radiosensitivity. Additionally, we found that lncRNAs competitively bind to miR-182 within the ceRNA network, influencing TUT4 target gene regulation. Together, these findings uncover critical molecular pathways underlying TUT4’s radioprotective function and suggest its potential as a therapeutic target for preventing radiation-induced esophageal injury. A key limitation of this study is the exclusive use of an animal model, without validation in cellular systems. Furthermore, biochemical mechanisms of TUT4 function remain to be elucidated through wet-lab experiments. Future studies will incorporate *in vitro* and *in vivo* approaches to validate these findings and further define the molecular mechanisms of TUT4-mediated radioprotection.

## Conclusions

Our study provides experimental evidence that TUT4 confers protection against radiation-induced esophageal injury. RNA-seq analysis revealed pathways and molecular mechanisms underlying TUT4-mediated radioprotection. Notably, TUT4 knockout esophageal tissues exhibited significant enrichment of the lipid metabolism, PPAR signaling, and RAS pathways, suggesting that TUT4 may exert its protective effects by repressing these radiation-responsive pathways, thereby enhancing radiosensitivity. Moreover, we identified a competing endogenous RNA (ceRNA) network in which lncRNAs sequester miR-182, modulating the expression of TUT4 target genes. This finding offers novel insights into the poorly understood role of miR-182 in radiation-induced esophageal damage. Bias from some unmeasured clinical variables may have weakened the validity of our study. In the future, we will verify our results in a follow-up in clinical drug practice. Meanwhile, it could be therapeutically targeted without compromising tumor control.

## Data Availability

The original contributions presented in the study are included in the article/**Supplementary Material**. Further inquiries can be directed to the corresponding authors.

## Supplementary material

SUPPLEMENTARY FIGURE 1 KEGG pathway diagram-Renin angiotensin system.The red nodes are associated with upregulated genes, and the duck green nodes are associated with downregulated genes.

SUPPLEMENTARY FIGURE 2 KEGG pathway diagram-PPAR signaling pathway diagram. The red nodes are associated with upregulated genes, and the duck green nodes are associated with downregulated genes.
