# Supplementary figures and images for: Potential protective regulatory effects on radiation−induced esophageal injury in TUT4−/− mice
Source: Front Oncol. 2025 Aug 26;15:1600597. doi: 10.3389/fonc.2025.1600597 (PMC12417170; doi:10.3389/fonc.2025.1600597)

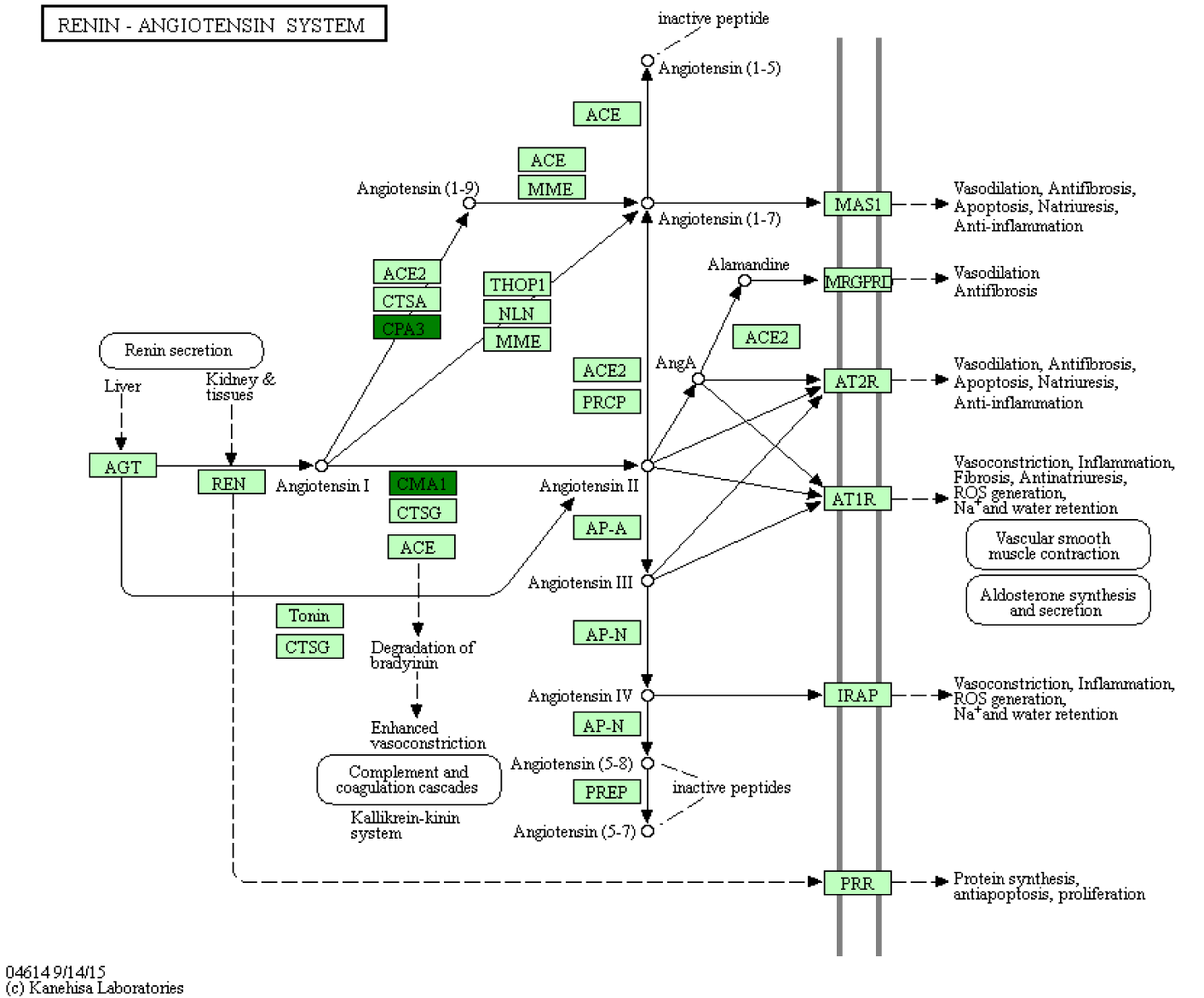

Supplement: SUPPLEMENTARY FIGURE 1 — KEGG pathway diagram-Renin angiotensin system.The red nodes are associated with upregulated genes, and the duck green nodes are associated with downregulated genes. [file Image1.tif]

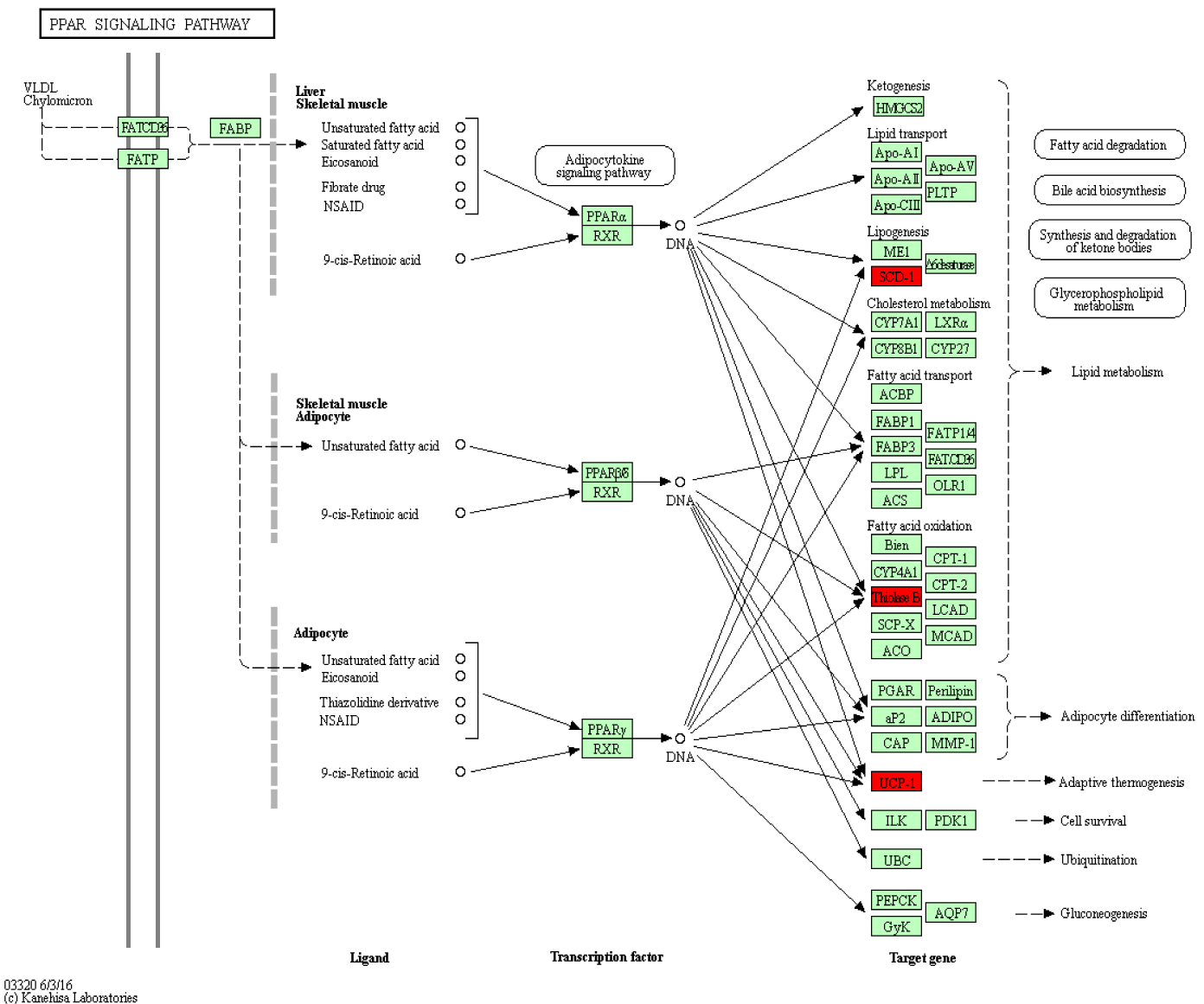

Supplement: SUPPLEMENTARY FIGURE 2 — KEGG pathway diagram-PPAR signaling pathway diagram. The red nodes are associated with upregulated genes, and the duck green nodes are associated with downregulated genes. [file Image2.tif]
